# Editorial: Assessing Microglial Function and Identity

**DOI:** 10.3389/fimmu.2021.824866

**Published:** 2021-12-20

**Authors:** Amanda Sierra, Rosa Chiara Paolicelli

**Affiliations:** ^1^ Achucarro Basque Center for Neuroscience, Glial Cell Biology Lab, Leioa, Spain; ^2^ Department of Neuroscience, University of the Basque Country EHU/UPV, Leioa, Spain; ^3^ Ikerbasque Foundation, Bilbao, Spain; ^4^ Department of Biomedical Sciences, University of Lausanne, Lausanne, Switzerland

**Keywords:** microglia, methods, central nervous system (CNS), disease, neuroimmunology and neuropathology

Microglia are the immune cells of the central nervous system. They are, however, a unique type of macrophage, as they arise from primitive myeloid progenitors in the embryonic yolk sac and do not repopulate from bone marrow-derived monocytes (1–3). In addition, they are instructed by brain activity and their highly motile processes contact all elements of the brain parenchyma (4–6). Over the past decades, a number of novel functions besides their classical immunological roles have been discovered, underscoring the importance of these cells in health and disease (7).

The field of microglia research is evolving at a rapid pace due to the equally fast development of both *in vitro* and *in vivo* methods and methodologies to label, visualize, and manipulate them. These tools include novel antibodies, viral vectors and transgenic mice specifically targeting microglia in invertebrate, rodent and primate models, as well as imaging tools for longitudinal analysis in humans. Validation of *in vitro* models of human microglia, including cells generated from induced pluripotent stem cells and organoids, will also push the field towards translational applications. Another important front is the development of drugs and nanomaterials that allow the selective manipulation of specific microglial functions. Finally, computational and modeling approaches, alongside with integrative –omics (epigenetic, transcriptomic, proteomic, and metabolomic) will come to maturity and help us understand the impact of microglia in brain physiology.

In this Research Topic we have collected 15 manuscripts, including original papers as well as literature reviews, that discuss the major methods currently available to investigate different aspects of microglial biology. These papers focus on methods to culture mouse and human microglia (Hernández et al., Hedegaard et al., Reich et al.) and to study microglia *in vivo* using live imaging tools (Eme-Scolan and Dando, Andoh and Koyama). Another set of papers pay attention to the microglial state characterized by transcriptomic and proteomic studies (Hsiao et al., Glasnović et al. , Miedema et al., Alsema et al.). Finally, a series of papers deal with microglial biology, including the study of autophagy (Plaza-Zabala et al.); and microglial function, including their role in synapse remodeling (Brioschi et al., Morini et al.) and as therapeutic targets in disease (Chen et al., Liu et al., Aires et al.).

*In vitro* and *ex vivo* methods are essential for investigating specific aspects and properties of microglial biology. Hernández et al. describe a novel protocol for inducing microglial reactivity to protein extract of injured rat spinal cord, and provide a comprehensive characterization, including morphology, phagocytic capacity and cytokine profile. Major efforts have been recently put into the development of human-induced Pluripotent Stem Cell (hiPSC) models to derive microglia from patients. The relevance of such models for investigating human diseases is unquestioned; however, the establishment of related protocols and functional assays is often challenged by reproducibility and scalability issues. Hedegaard et al. review the current methods available to generate hiPSC-derived microglia and discuss areas for improvement, advantages and limitations. By establishing a new type of hiPSC-derived microglia, Reich et al. investigate the regulatory role of TREM2, and provide a detailed characterization of the functional microglial properties affected by TREM2 loss of function.

Microglia are extremely sensitive to external stimuli and able to rapidly change their state in response to perturbations in the brain environment. This remarkable ability of microglia calls for the need of developing more and more advanced techniques and tools for studying them *in situ*, and catching their dynamic behavior. Eme-Scolan and Dando provide an overview on the current tools and approaches for studying microglia *in vivo*, highlighting advantages and limitations. In detail, Andoh and Koyama review different experimental systems available for live imaging and discuss some of the molecular mechanisms underlying microglial dynamics.

Insights into relevant molecular pathways regulating microglial states are provided in two distinct reviews, which systematically analyze publicly available transcriptomic data, to discuss the role of G-protein-coupled-receptors (GPCRs) and the RANKL/RANK/OPG (RRO) axis (Hsiao et al., and Glasnović et al., respectively). Both works highlight the therapeutic potential of these signaling pathways for targeting microglial dysfunction in brain disease. A number of studies in this collection examine the emerging roles of microglia in pathology, discussing the microglial profile, by complementary angles, in a variety of neurodegenerative disorders. Miedema et al. highlight the importance of microglial heterogeneity, reviewing high-resolution transcriptomic and proteomic approaches to assess microglia in multiple sclerosis. Using acute human *post-mortem* brain samples, Alsema et al. investigate the microglial transcriptome of Alzheimer’s disease (AD) and non-demented (ND) elderly, reporting a core similarity between AD and ND donors.

Understanding microglial physiology is essential to appraise their role from development to aging and disease. One key process is autophagy, which is in charge or recycling intracellular organelles. Plaza-Zabala et al. propose a two-step model to assess autophagy in microglia, and offer a detailed method to determine autophagosome formation, degradation and net turnover, which will help understand the role of autophagy in healthy and diseased microglia. Microglia-mediated synapse elimination has received growing attention in the last years, both in physiological and pathological contexts. Novel methodological approaches are continuously developed to meet the need for accurate investigation and quantification of this process. Brioschi et al. propose a novel technical approach for the assessment of synaptic engulfment by microglia, based on flow cytometry, and provide quantification of synapse engulfment in microglia acutely isolated from the developing mouse brain, and from the brain of mouse models of neurodegeneration. The current methods available to investigate microglial phagocytosis of neuronal and synaptic structures are extensively reviewed by Morini et al., who focus on critical developmental time windows, and discuss the implication of specific ligand-receptor crosstalk.

Further implications of microglia in the diseased brain are discussed by Chen et al., with a special focus on neuromyelitis optica (NMO), an autoantibody-triggered disease affecting the spinal cord and the optic nerve. Liu et al. examine the complex roles of microglia in intracerebral hemorrhage, revising the distinct phenotypes and dynamic profiles associated with functional responses upon the hemorrhagic event. Age-related impairments in microglial function are discussed by Aires et al., who provide evidence for CD22 blockage as a potential therapeutic approach for restoring microglial ramification and surveillance capacity, notably reduced in the ageing brain.

Taken all together, the manuscripts gathered in this Research Topic strongly emphasize the heterogeneity and complexity of microglia, and their multifaceted roles in the healthy and diseased CNS. These studies also highlight the undisputed importance of carefully reporting the spatial and temporal coordinates when describing microglial profiles and associated functions, to facilitate data integration and interpretation across studies.

In conclusion, we hope this collection of systematic reviews and original methods may serve as a reference for the field, and may promote a culture of exchange for synergistically improve the current techniques available to assess microglial identity and function.

## Author Contributions

AS and RCP both contributed to this editorial by resuming the results of all scientific manuscripts included in the Research Topic. All authors contributed to the article and approved the submitted version.

## Funding

This work was supported by the Spanish Ministry of Science and Innovation CompetitivenessMCIN/AEI/10.13039/501100011033 (https://www.ciencia.gob.es/) and FEDER “A way to make Europe” (RTI2018-099267-B-I00 and RYC-2013-12817), a Tatiana Foundation Award (P-048-FTPGB 2018), and a Basque Government Department of Education project (PIBA 2020_1_0030;http://www.euskadi.eus/basque-government/department-education/) to AS; by grants from the Synapsis Foundation - Alzheimer Research Switzerland ARS, the Swiss National Science Foundation (SNSF 310030_197940) and the European Research Council (ERC StGrant REMIND 804949) to RCP.

## Conflict of Interest

The authors declare that the research was conducted in the absence of any commercial or financial relationships that could be construed as a potential conflict of interest.

## Publisher’s Note

All claims expressed in this article are solely those of the authors and do not necessarily represent those of their affiliated organizations, or those of the publisher, the editors and the reviewers. Any product that may be evaluated in this article, or claim that may be made by its manufacturer, is not guaranteed or endorsed by the publisher.
